# Endonasal endoscopic or endoscopic-assisted transcranial surgery of Rathke’s cleft cysts: does the approach and surgical technique influence the radicality and recurrence rate?

**DOI:** 10.1007/s10143-024-02545-3

**Published:** 2024-08-05

**Authors:** Stefan Linsler, Laura Schon, Gerrit Fischer, Sebastian Senger, Joachim Oertel

**Affiliations:** Department of Neurosurgery, Faculty of Medicine, Saarland University Medical Center and Saarland University, Homburg, Germany

**Keywords:** Rathke´s cleft cyst, Endoscopy, Keyhole approach, Endonasal skull base surgery, Endoscopic assisted surgery

## Abstract

Objective: Resections of symptomatic Rathke’s cleft cysts (RCCs) are mainly performed via an endonasal transsphenoidal approach. However, there is a lack of equivalent data in current literature concerning transcranial keyhole approach in the treatment of RCCs. In order to find general recommendations for the surgical treatment of RCCs also with regard to recurrence, the object of this study is the analysis and comparison of both techniques. Methods: Twenty-nine patients having been surgically treated between January 2004 and August 2019 were retrospectively analysed. The transsphenoidal approach was chosen in 16 cases and the transcranial keyhole approach in 13 cases. Both surgical techniques were analyzed and compared concerning preoperative symptoms and cyst characteristics, complications, surgical radicality, endocrinological and ophthalmological outcome and recurrences in patients´ follow up. Results: The postoperative outcome of both techniques was identic and showed highly satisfying success rates with 92% for neurological deficits, 82% for endocrinological dysfunctions and 86% for visual deficits. In contrast, momentous postoperative complications were significantly more likely after transsphenoidal operations. After a mean follow-up time of 5.7 years, the recurrence rates of both cohorts were the same with 0% each. Conclusions: Regarding its equal outcome with its lower complication rate, the authors suggest using the supraorbital keyhole approach for RCCs whose anatomical configuration allow both techniques. Yet, the decision should always consider the surgeon’s personal experience and other individual patient characteristics. Further studies with higher numbers of cases and longer follow-up periods are necessary to analyse the effect of the selected approach on recurrence.

**Supplementary Information:**

The online version contains supplementary material available at 10.1007/s10143-024-02545-3.

## Introduction

Rathke’s cleft cysts (RCCs) are benign cystic remnants of the embryological development of the anterior pituitary that are incidentally found in 5–33% of autopsies [1, 2]. Unfortunately, it is often difficult to distinguish RCCs from other neoplastic lesions during diagnostic investigation because of a wide variability of appearance in radiological imaging [3]. They usually show an intra- and suprasellar extension in up to 95%, but purely intrasellar or suprasellar cysts occur as well [4, 5]. Also, they might appear as hypo-, iso- or hyperintense in MR imaging as well as hypo-, iso- or hyperdense in CT imaging [3]. Due to their location in the sellar region, typical reasons for demanding medical advice are endocrinological dysfunction in 17–81%, headache in 44–93% and visual deficits by affection of the optic chiasm in 11–67% [6–9]. Whilst asymptomatic patients without cyst growth must not be treated surgically in every case but followed at regular intervals, there are two common surgical techniques for symptomatic cases in order to reach the hypophyseal fossa: the endoscopic endonasal approach and the endoscopic-assisted transcranial keyhole approach [10, 11]. Based on a high level of experience and low complication rates, the endonasal technique is the first choice in most neurosurgical centres for treatment of pathologies in the sellar region [6, 12–20]. Modern technical innovations in endoscopic endonasal skull base surgery like HD-endoscopy or angled optics allow minimal-invasiveness with highly satisfying outcomes and low complication rates as described before [16, 17]. Main advantages of the endonasal approach are the direct access to the sellar region without brain retraction and a straight forward procedure [18, 21, 22]. Major drawbacks are the increased risks for postoperative CSF fistulas, sinonasal complications and the limited access to lateral structures [11, 14, 17, 19, 23, 24].

In contrast, the transcranial keyhole technique, mostly performed via supraorbital minicraniotomy, is usually reserved for suprasellar RCCs that are difficult to be reached via endonasal approach [8, 11]. Benefits of this technique are a bigger range of motion in the surgical field and a minor manipulation of the pituitary gland, whereas brain retraction and lesions of the cranial nerves I, II, V and VII are listed as main risks [11, 25–28]. Postoperative cyst recurrence was reported in up to 43% of cases in literature and the promoting factors are still object of research [10, 24, 29, 30]. Thereby, there is still a debate about the different surgical techniques and the surgical aim focusing on maximal gross total resection of only a cyst fenestration with suction of the fluid content.

Considering the advantages of the transcranial keyhole approach, the authors’ aim was to evaluate whether its use might help to improve outcome, reduce complication rates or even decrease the risk of cyst recurrence in comparison to the commonly used endonasal approach. Thus, this study should help to develop recommendations for surgical treatment of Rathke’s cleft cysts.

## Materials and methods

### Study design, patient population and imaging studies

In this study, all 24 patients undergoing surgical treatment for RCC from February 2011 to August 2019 at the Department of Neurosurgery, Saarland University, Germany, were retrospectively reviewed. Five more patients having been treated between January 2004 and November 2007 at the Department of Neurosurgery, Klinikum Nordstadt, Hannover, Germany, were added to the patient cohort.

Inclusion criteria were a complete set of preoperative and postoperative images, the diagnosis of a RCC by the the presence of a non-neoplastic epithelial cyst wall and PAS-positive amorphous substance, a complete patient record, including complete postoperative follow-up, and the presence of a detailed video recording of the surgical procedure.

Surgical treatment was indicated in case of cyst-related symptoms, laboratory evidence of pituitary dysfunction, cyst growth or unclear diagnosis in preoperative imaging. The patient cohort was subdivided in two groups: group A included all patients having undergone transcranial surgery, in which all interventions were performed via supraorbital keyhole approach. Group B included all patients having undergone endonasal transsphenoidal approach. Thereby, the cases with opening of the diaphragm to intracranial during surgery were analysed separately in this cohort of group B.For data collection, all available documents were reviewed including medical records, surgical and histopathological reports, video recordings, clinical visits and pre- and postoperative imaging studies. The retrospective study was authorized by the ethical committee of the medical association of the Saarland (Nr. 44/21).

Our clinical study focused on analyzing data pertaining to cyst and patient characteristics, surgical procedures, preoperative symptoms, postoperative outcomes, complications, and recurrence. Endocrinological symptoms, outcomes, and complications were assessed through serological testing of hormone levels, including morning cortisol, ACTH, TSH, GH, IGF-1, LH, FSH, and prolactin. Visual function was assessed through visual field and visual acuity testing, while neurological symptoms and outcomes were evaluated based on thorough patient examinations. All patients underwent pre- and postoperative magnetic resonance imaging (MRI) with Gadolinium as the contrast agent. Follow-up included routine MRI studies every 12 months. Tumor volume was determined by measuring the longest diameter laterally and from rostral to occipital in the axial plane, along with the longest axial diameter shown in coronal T1-weighted images enhanced with Gadolinium contrast. Additionally, CT imaging was conducted before surgery to plan the surgical approach and after surgery to assess the extent of resection. One patient underwent only CT imaging due to having a pacemaker.

### Selection of the surgical approach

Important factors for the preoperative selection of approach were cyst localisation and cyst configuration, the surgeons’ personal experience and the suspected diagnosis. All procedures were performed by a senior neurosurgeon (JO, SL) with experience in both techniques: endonasal endoscopic approaches and keyhole transcranial approaches to sellar region.The selection of the endonasal or supraorbital route was determined by the preference of the operating neurosurgeon, taking into account factors such as tumor size, localization, and invasiveness into structures specific to each case.

### Surgical technique

**Fig. 1:**
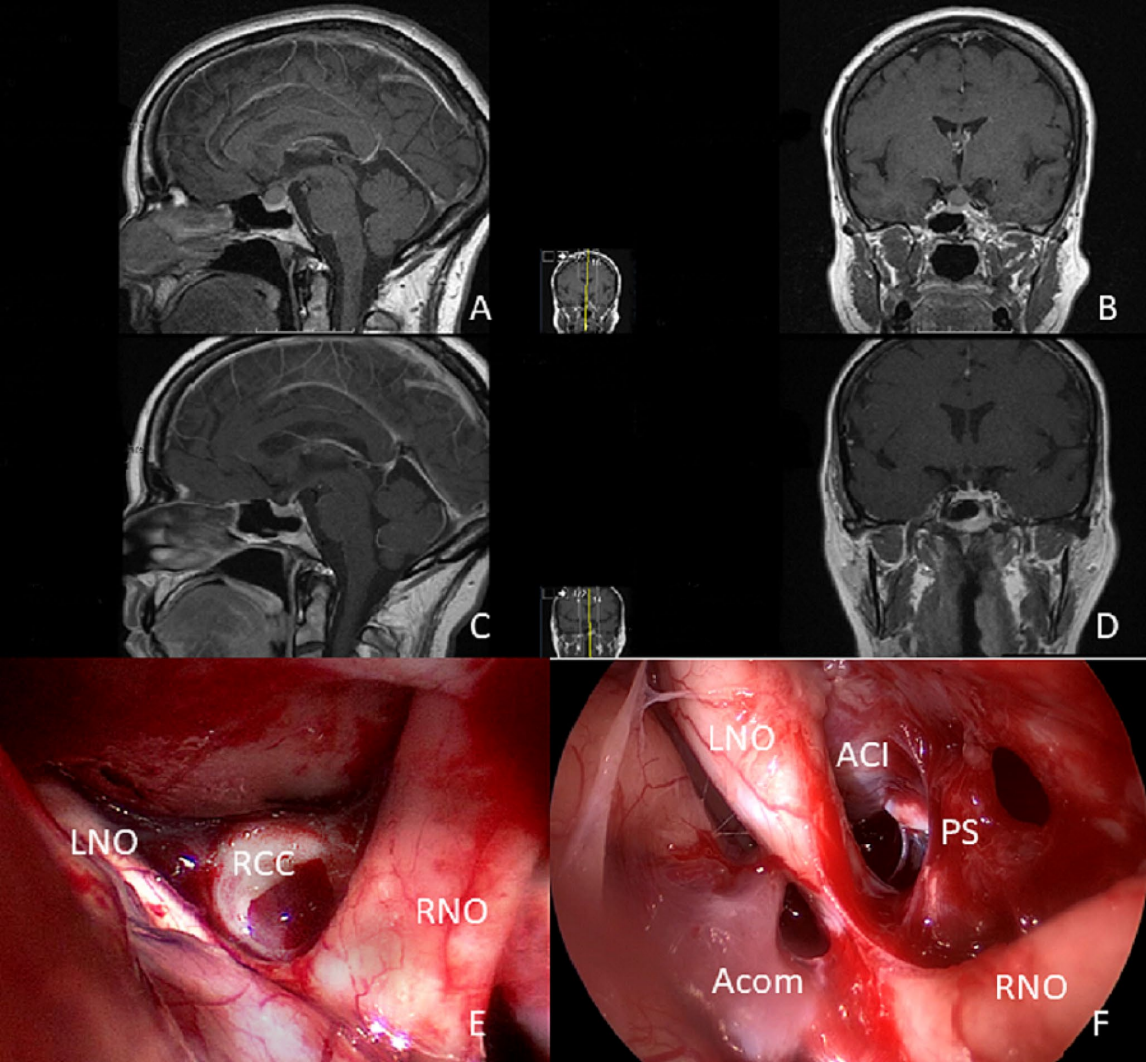
Illustrative case of a 38-year-old woman with headache and diplopia. The MRI scan showed a 13 × 12 × 11 mm cystic lesion suprasellar with compression of the pituitary gland in sagittal **(A)** and coronal **(B)** imaging. Postoperative MRI showed no remnant cyst tissue (**C**, **D**). Gross total resection was achieved by using a supraorbital keyhole craniotomy with endoscopic assistance. **E**: microsurgical view of the surgical field after opening of the RCC (LON: left optic nerve, RON: right optic nerve, RCC: Rathke´s cleft cyst). **F**: intraoperative inspection of supra- and intrasellar region with 30° endoscope (LON: left optic nerve, RON: right optic nerve, PS: pituitary stalk, Acom: anterior communicating artery, ACI: left internal carotid artery). There was no remnant cyst tissue visualized at the end of resection with the endoscope

The authors have described their transcranial as well as the transsphenoidal surgical approaches and techniques in previous reports [12, 27]. The surgical approach via keyhole craniotomy with supraorbital skin incision was performed as previously described in other reports [27, 31, 32] with a frontolateral minicraniotomies (size approximately 2.5 × 1.5 cm).The endoscopic devices with rigid rod lens endoscopes with outer diameters of 4.0 mm and different angles of 0°, 30°, and 70° (Karl Storz, Tuttlingen, Germany), were accessible throughout the whole surgical procedure. The utilization of an endoscope was indiciated by the neurosurgeon if it deemed beneficial.The neurosurgeon primarily handled the endoscope during the procedure, with a holding device available for assistance. The endoscope’s efficacy was gauged by its capability to detect residual pathological tissue that may not be visible under the microscope, ensuring thorough resection intraoperatively. Additionally, it aided in the precise removal of pathological tissue under endoscopic guidance, contributing to the overall success of the surgery The cyst resection involved incising the cyst wall and aspirating its contents. The cyst wall was carefully removed without causing damage to the pituitary gland or surrounding structures.(see Fig. 1 with an illustrative case and suppl. Video 1).

The endoscopic endonasal surgical technique in the sellar region were performed by the authors as previously described in larger series [12, 18]. All procedures were performed without an ENT surgeon in the OR.

**Fig. 2:**
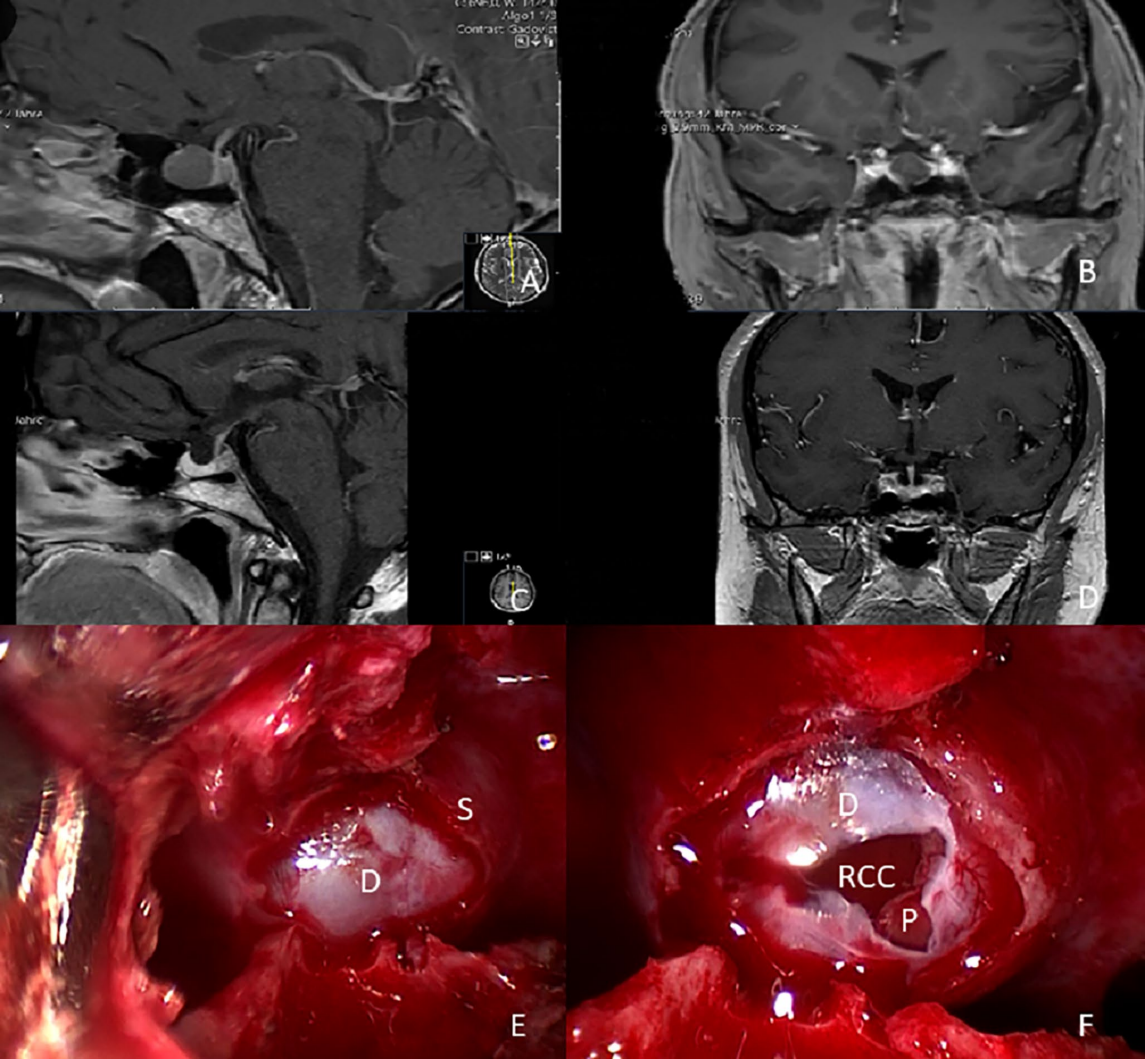
Illustrative case of a 55-year-old woman with since 3 months hormonal insufficiency. The MRI scan showed an intrasellar cystic lesion in front of the pituitary gland (12 × 14 × 11 mm) in sagittal **(A)** and coronal **(B)** imaging. In postoperative MRI scan no remnant tumor tissue was detected (**C**, **D**). Endoscopic view of the sellar region before opening of the outer dura layer and cyst wall **(E)** and endoscopic intrasellar inspection after cyst opening **(F)**. (S: sella, D: outer dura layer, P: pituitary gland, RCC: opened Rathke´s cleft cyst)

During endonasal surgery, both lateral fluoroscopy (C-arm) and MRI-based neuronavigation were routinely utilized for intraoperative orientation and imaging. The approach and cyst removal were conducted using either 4 mm–2.7 mm rigid endoscopes with Hopkins optics and 0°-angled lenses (Karl Storz, Tuttlingen, Germany). Subsequently, scopes with 30°- and 70°-angled lenses were employed for final inspection to enhance radicality and facilitate resection of pathologies. The cyst resection involved incising the cyst wall and aspirating its contents. The cyst wall was carefully removed without causing damage to the pituitary gland or surrounding structures. If there was an intraoperative CSF leakage and dural opening, closure was consistently performed using a sandwich technique with an autologous periumbilical fat graft in an underlay technique, with the periumbilical abdomen appropriately prepared for graft harvesting in combination with Tachosil^®^. Additionally, after surgery lumbar drainage was inserted, and skull base defect reconstruction involved the use of bone pieces, while fibrin glue and Tachosil^®^ for the sellar floor reconstruction.Novascularized nasoseptal flap was utilized in any of these cases (see Fig. 2 with an illustrative case and suppl. Video 2). All procedures were video recorded and analysedGross total resection (GTR) was defined as resection of all loose cyst parts. Near total resection (NTR) was defined as > 80% resection and subtotal resection (STR) as ≤ 80% resection of the lesion or removal of cyst content and partial cyst wall resection.

### Clinical outcome and follow-up

Complications were thoroughly analysed by reviewing the surgical report, video recordings, medical reports, postoperative imaging, and follow-up data. For assessing pituitary function, serological testing of hormone levels including morning cortisol, ACTH, TSH, GH, IGF-1, LH, FSH, and prolactin was conducted both pre- and postoperatively. Additionally, urine specific gravity was routinely analyzed after surgery. To evaluate the visual function postoperatively, all patients underwent comprehensive visual function assessments, including formal visual field testing.

The patients were regularly examined at the neurosurgical outpatient department of Saarland University as well as in the endocrinological department for routine follow-up or in response to the emergence of neurological symptoms. Recurrence were determined by identifying new evidence of tumor on MR imaging studies, evaluated by a neuroradiologist after previous GTR.

### Statistics

The analysis and visualization of data were conducted using IBM^®^ SPSS^®^ Statistics Version 26 (SPSS Inc., Chicago, USA). Patient cohorts were compared using the Whitney-U-Test, Fisher’s exact test, and multivariate analysis to assess differences between values in the groups. Significance level was set at *p* < 0.05.

## Results

### General results and demographic data

A total of 29 patients with histologically confirmed Rathke’s cleft cyst were included in the retrospective analysis. The transcranial keyhole approach was performed in 13 cases (45%, Group A), whereas the endoscopic endonasal resection was chosen in 16 cases (55%, Group B). The patients’ average age was 52 years [19–86, SD ± 18 years] and there were 19 females (66%) and 10 males (34%). Mean follow-up time was 70.2 ± 31.5 months. All demographic data were comparable amongst Group A and B (see Table 1).

**Table 1:** Characteristics of patient cohorts. Significant levels (*p* < 0.05) are assigned (*)

	All (*n* = 29)	Group A (transcranial) *n* = 13	Group B (endonasal) *n* = 16
**Demographic data**			
**mean age [yrs]**	52 (19–86)	52 (28–68)	51 (19–86)
**w: m [%]**	66:34	69:31	63:37
**RCC characteristics**			
**mean volume [cm³]**	2,1 (0,1–12,0)	1,1 (0,4 − 2,8)*	2,7 (0,1–12,0)
**squamous metaplasia**	3% (*n* = 1)	0% (*n* = 0)	6% (*n* = 1)
**Localisation**			
**suprasellar**	11% (*n* = 3)	23% (*n* = 3)*	0% (*n* = 0)
**Intrasellar**	32% (*n* = 9)	8% (*n* = 1)	53% (*n* = 8)*
**supra- and intrasellar**	57% (*n* = 16)	69% (*n* = 9)	47% (*n* = 7)
**Preoperative complaints**			
**headache**	28% (*n* = 8)	23% (*n* = 3)	31% (*n* = 5)
**visual deficits**	48% (*n* = 14)	69% (*n* = 9)	31% (*n* = 5)
**hormonal insufficiency**	24% (*n* = 7)	23% (*n* = 3)	25% (*n* = 4)
**hyperprolactinaemia**	17% (*n* = 5)	15% (*n* = 2)	19% (*n* = 3)

### Preoperative symptoms

23 patients (79%) showed preoperative symptoms which lead to medical consultation, whilst in 6 cases (21%), the cyst was discovered incidentally. In the course of diagnostics then, pathological irregularities were detected in 27 patients (93%). One-third (31%) of them experienced multiple symptoms at the same time. Preoperative symptoms included visual deficiency, endocrinological dysfunction and neurological symptoms. Visual deficiencies, in detail visual field loss and loss of vision, were the most common symptoms with 48% (*n* = 14). They occurred more often in group A with 69% (*n* = 9) than in group B with 31% (*n* = 5). 93% (*n* = 13) of the patients suffering from visual deficiency had an entirely or partially suprasellar cyst configuration. Neurological symptoms, including headache, dizziness, reduced consciousness, nausea and emesis occurred in 41% (*n* = 12). They were more common in group B with 50% (*n* = 8) than in group A with 31% (*n* = 4). Headache as the most important neurological symptom was reported by 28% (*n* = 8) of the patients. Endocrinological dysfunction, including hyperprolactinaemia and pituitary insufficiency, was found in 38% of the patients (*n* = 11). It was more common in group B with 43% (*n* = 7) than in group A with 31% (*n* = 4) as well. For an overview of the most presented complaints see Table 1.

### Cyst characteristics, resection radicality and surgical details

Average cyst volume was significantly bigger in group A (2,7 cm³) versus group B (1,1 cm³) (*p* < 0.05), and squamous metaplasia was found only in one patient of group B. Concerning cyst localization, both groups differed significantly (*p* < 0.05). In group B, supra- and intrasellar cysts (47%, *n* = 7) and strictly intrasellar cysts (53%, *n* = 8) were found in about half of the patients each, but no strictly suprasellar cyst occurred. In group A, the majority of cysts was intra- and suprasellar (69%, *n* = 9). Strictly suprasellar cysts were found in three patients (23%) and only one strictly intrasellar cyst (8%) occurred. On the other hand, all strictly suprasellar cysts (100%, *n* = 3) were resected via transcranial approach and all strictly intrasellar cysts except one (89%, *n* = 8) were resected via transsphenoidal approach. Supra- and intrasellar cysts were resected via transcranial approach in 56% (*n* = 9) and via transsphenoidal approach in 44% (*n* = 7). Mean surgical time in group A was with 119 min (87–179 min) nearly twice as long as in group B with 65 min (32–92 min) (*p* < 0.01). GTR was significantly more frequent in group A with 85% (*n* = 11) than in group B with 31% (*n* = 5) (*p* = 0,004). Thereby, the diaphragm was opened intraoperatively in 56% (9/16) in case of an endonasal approach. During cyst resection, intact gland tissue and pituitary stalk was mostly identified either at the beginning of resection or after gross total removal at final endoscopic inspection. Intact vital pituitary tissue (exemplary image is shown in Fig. 2F) was identified in 69% (9/13) in the transcranial group and in 87% (*n* = 14) in the endonasal group. The pituitary stalk was identified in 100% (13/13) in the transcranial group and in 50% (*n* = 8) in the endonasal group (*p* < 0.05) intraoperatively by the surgeon (see Table 2). In all cases, the intraoperative localization of the gland corresponded to expected localisation based on preoperative imaging.

**Table 2:** Details of intraoperative findings and surgical outcome of both cohorts of the study (GTR = gross total resection, NTR = near total resection, STR = subtotal resection)

	Group A (transcranial) *n* = 13	Group B (endonasal) *n* = 16	
**Radicality**			
**GTR**	85% (11)	31% (5)	*P* < 0.005
**NTR**	15% (2)	31% (5)	*P* < 0.05
**STR**	0%	38% (6)	*P* < 0.005
**Recurrence rate**	0%	0%	
**Opening of the diaphragm**			
	100% intradural	56% (*n* = 9)	
**Identification of**			
**Pituitary gland**	69% (*n* = 9)	87% (*n* = 14)	
**Pituitary stalk**	100% (*n* = 13)	50% (*n* = 8)	*P* < 0.05

In 56% (9/16) cases of the endoscopic endonasal resection of a RCC an intraoperative opening of the diaphragm occurred with consecutive intraoperative CSF flow. In 8 of these cases a closure in sandwich technique with autologous fat graft, fascia lata and Tachosil^®^ was performed as described previously by the authors. Additionally, the authors applied a lumbar drainage for 5 days in these 8 cases. In one case with only minimal CSF flow intraoperatively, the dural closure was performed with Tachosil^®^ and fascia lata only. Details are illustrated in Table 3.

**Table 3:** Technique of dural reconstruction according to CSF leakage intraoperatively after resection of RCCs (*n* = 9)

Intraoperative CSF Flow (Group B)	Tachosil	Fascia lata/ Duragen	Autologous fat graft	Lumbar drainage	vascularised mucosal flap
No (*n* = 8)	-	-	-	-	-
Minimal (*n* = 1)	1	1	0	0	-
Yes (*n* = 8)	8	8	8	8	-

### Application of the endoscope

In all cases, the endoscope was utilized. Thereby, the rigid 0° rod lens Hopkins optic was employed for all endonasal procedures.In 10 out of 16 cases (62.5%), a 30° endoscope was used additionally. No emergency stops or transitions to microsurgery occurred during the procedures.Whenangled optics were employed at the end of resection procedure, remnant tumor tissue was identified in 5 out of 10 cases (50%), as demonstrated in Table 4. The endoscope was utilized to inspect the suprasellar space in 6 cases, leading to the visualization of remnant cyst wall in 3 cases, and intrasellar space in 10 cases, with remnant cyst wall identified in 3 cases. Further details are provided in Table 4.

**Table 4:** Endoscopic inspection of the surgical field according to the identification of remnant tumor tissue in the different localizations

**Supraorbital approach:**		
	**Endoscopic inspection after cyst removal (** *n* ** = 13)**	**Identification of remnant tissue**
Sellar space	13 (100%)	6/13 (46%)
Optocarotid window	6 (46%)	1/6 (16%)
Retrochiasmatic space	3 (23%)	1/3 (33%)
**Endonasal approach:**		
	**Endoscopic inspection with angled scope after cyst opening and removal (** *n* ** = 10)**	**Identification of remnant tissue**
intrasellar space	10 (100%)	3/10 (30%)
suprasellar space	6 (60%)	3/6 (50%)

In all cases involving the transcranial approach, the endoscope was utilized to inspect the surgical resection area (*n* = 13).In each case, the neurosurgeon found the endoscope beneficial, assisting in the identification of remnant cyst tissue, pituitary gland tissue, and the pituitary stalk, as well as ensuring total resection. Remnant cyst tissue was detected with angled optics in 46% (6/13) of all cases, as detailed in Table 4. The endoscope was employed to explore the opticocarotid window in six cases, resulting in the identification of remnant cyst tissue in one case. Furthermore, it was utilized to examine the sellar space in 13 cases, aiding in the visualization of the diaphragm sellae, gland tissue, and remnant cyst tissue, with remnant tissue identified in six cases. Additionally, the endoscope was used to inspect the retrochiasmatic space in three cases, with remnant tissue found in one case. Detailed findings are presented in Table 4.There were no intraoperative complications or technical issues associated with the use of the endoscope.

### Postoperative outcome

With 78% (*n* = 21) of the symptomatic patients experiencing a complete relief of their deficiencies and 7% (*n* = 2) experiencing an amelioration, a total of 85% of symptomatic patients (*n* = 23) benefitted directly from surgical intervention, whereas 15% (*n* = 4) did not show any improvement. The postoperative outcome of both techniques revealed a satisfying success rates without significant differences. Furthermore, there was not significant difference between completely and partially resected cysts in the postoperative outcome. Details are demonstrated in Table 5.

**Table 5:** Postoperative outcome of the preoperative complaints

All (*n* = 29) Preoperative complaints	improvement	unchanged	worsening
**visual deficits (** *n* ** = 14)**	85% (12/14)	15% (2/14)	0%
**headache (** *n* ** = 8)**	87% (7/8)	13% (1/8)	0%
**endocrinological dysfunction (** *n* ** = 12)**	75% (9/12)	25% (3/12)	0%

Amongst the fourteen patients with visual deficiencies, 79% (*n* = 11) reported a complete relief, 6% (*n* = 1) reported an amelioration, and 15% (*n* = 2) did not improve. In detail, loss of vision improved in 100% (*n* = 9) and visual field loss in 78% (*n* = 7). Amongst the twelve patients with neurological symptoms, 92% (*n* = 11) benefitted from the intervention. In detail, 87% (*n* = 7) of patients with headaches reported a complete relief, whereas one patient (13%) did not improve. All other neurological symptoms disappeared in 100% (*n* = 7). Amongst the twelve patients with endocrinological dysfunction, 67% (*n* = 8) reported complete relief, 8% (*n* = 1) reported amelioration, and 25% (*n* = 3) did not improve. In detail, hyperprolactinaemia disappeared in 100% (*n* = 6) and pituitary insufficiency in 57% (*n* = 4). The cases of pituitary insufficiency with no improvement (*n* = 3) had a distinct preoperative impairment of the pituitary gland with affection of three or more hormonal axes in common.

The recurrence rate of both cohorts were the same with 0% after mean follow-up of 5.7 years.

### Complications

**Fig. 3:**
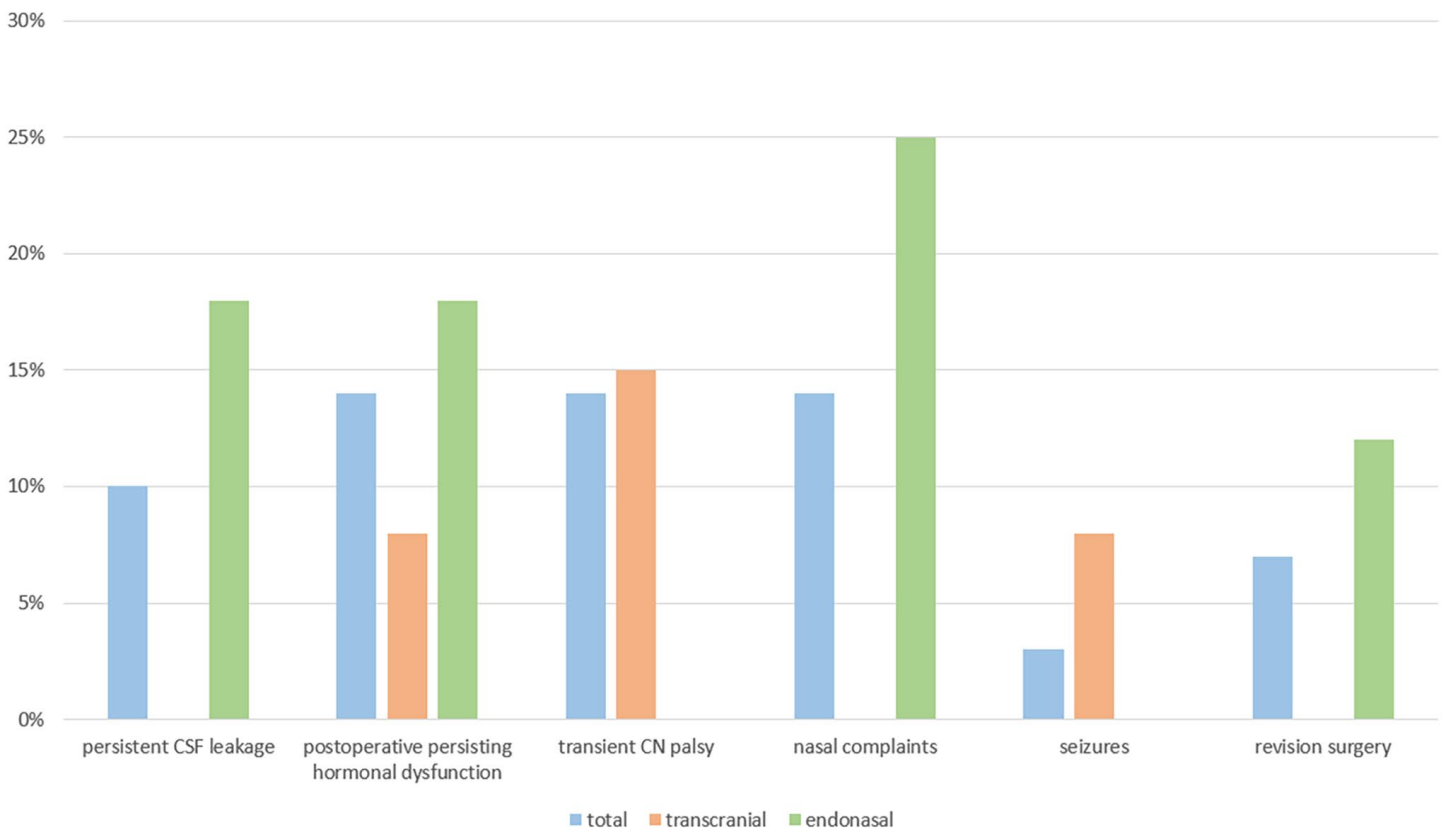
Complications rate [%] of both cohorts and of all patients in detail

Intraoperative and postoperative complications were subdivided in general and approach related complications. Overall, their occurrence was not associated with the extent of resection, the configuration, or the size of the cyst in the multivariate analysis. Among the general postoperative complications, there was transient hormonal dysfunction in form of diabetes insipidus in 24% (*n* = 7), which occurred more often in the transsphenoidal group with 38% (*n* = 6) than in the transcranial group with 8% (*n* = 1). In addition, permanent hormonal dysfunction in form of newly appeared or worsened pituitary insufficiency occurred in 14% (*n* = 4) and more often in the transsphenoidal group with 19% (*n* = 3) than in the transcranial group with 8% (*n* = 1). The insufficiencies affected the gonadal axis in three cases, the thyroid axis in two cases and the adrenal axis in one case. Besides, the one patient of the transcranial group already suffered preoperatively from pituitary insufficiency, which worsened after surgical intervention, whereas the three patients of the transsphenoidal group did not have any insufficiency beforehand. Other general complications were one case (3%) of postoperative singular seizure in the transcranial group without necessity of further treatment in the follow up. Approach related complications of the transcranial group were transient cranial nerve lesions in 15% (*n* = 2). These lesions caused one case of hyposmia by affection of the olfactory nerve, and one case of facial nerve palsy with affection of the temporal branch. Additionally, two patients presented with postoperative hypaesthesia by affecting of the supraorbital nerve. All patients recovered completely within one year during follow up. Approach related complications of the endonasal group were postoperative persisting CSF fistula in in 18.7% (3/16) and nasal complications in form of recurring sinusitis or affected breathing through the nose, which demanded further treatment in the ENT department in four cases (25%). The persisting CSF fistulas required resurgery and further duraplasty in two cases (12%). One of these two fistulas emerged from a CSF leakage that was not identified during initial surgery and therefore not closed, whereas the other fistula resulted from a bone piece having loosened from sphenoid plasty. One case was treated with further lumbar drainage for 6 days successfully. The mortality rate was 0%, and no severe or permanent procedure related complications occurred. The postoperative outcome and the complications are illustrated in Table 5; Fig. 3 in detail.

## Discussion

Rathke’s cleft cysts have a high prevalence and stay mostly asymptomatic [10]. Complaints usually appear through mass effect and compression of surrounding structures when the cyst starts growing [33]. In cases of symptomatic RCCs, there is wide consensus on the necessity of surgical treatment in the current literature [1, 6, 34, 35]. While permanent damages of the visual, olfactory or endocrinological system need to be prevented [6]. In most neurosurgical centres, those resections are routinely performed via a minimally invasive, transsphenoidal microscopic or endoscopic approach, since in the context of other neoplasia like meningioma or pituitary adenoma, most surgeons have a high level of experience with it [8, 12, 18, 19, 24, 30, 36]. However, the transcranial keyhole approach is in general limited to patients whose cyst morphology or other individual characteristics count against the transsphenoidal approach and is therefore used only in few, often complex cases actually [8, 11]. Due to this lack of equivalent data, the question whether the transcranial or the endonasal approach shows better radicality, recurrence rate und postoperative outcome remained so far unanswered. In order to find the technique with the best postoperative outcome but also with the lowest complication rate, the two patient cohorts of this study were compared with regard to their preoperative symptoms, surgical data, postoperative outcome and complications. Thereby, the main criterion of the neurosurgeon for choosing the approach was the cyst localisation in preoperative imaging. A cyst located at the bottom of the hypophyseal fossa is ideally approached through a transsphenoidal approach. In contrast, a completely suprasellar cyst is more readily accessed via a transcranial approach, avoiding manipulation of the gland tissue [11]. This principle helps to preserve the anatomical surrounding by choosing the shortest or most direct trajectory. Furthermore, the risk of postoperative new hormonal deficiencies should be minimized by a transcranial route [11, 25, 27]. Therefore, the aim of the authors’ study is to determine the optimal surgical approach in cases where both techniques are viable, as demonstrated in their retrospective analysis of 29 patients. Given the retrospective nature of our work and the aforementioned point, it is unsurprising that there were differences in cyst morphology between the two cohorts. Specifically, there were significantly more purely suprasellar cysts in the transcranial group, while all purely intrasellar cysts were resected via the transsphenoidal approach. This is in line with previous reports of the literature [11]. The percentages of the individual cyst locations were comparable with those reported in previous studies [1, 7, 37, 38]. The higher number of suprasellar cysts in the transcranial group was attended by a higher incidence of preoperative visual deficits, which is based on the suprasellar location of the optic chiasm. The transsphenoidal group in contrast contained more cases of endocrinological dysfunction, which can be explained by the fact, that all of the cysts had a purely or partly intrasellar location neighbouring the pituitary gland. In addition, neurological symptoms like headache or nausea were more numerous in the transsphenoidal group, which is probably due to its higher average cyst volume and a therefore distinct meningeal irritation.

In terms of postoperative outcome, the present study showed equality of the two analysed surgical techniques. Both approaches allowed an adequate partial removal of the cyst wall with NTR of 15% in the transcranial cohort and of 31% in the endonasal cohort. The GTR rate differed significantly (85% vs. 31%). However, the radicality did not influence the recurrence rate and the postoperative follow up. There was no recurrence in both cohorts within the long-term follow up (mean 5.7 years). Therefore, the authors stated, that the outcome did not depend on the extent of cyst wall resection, given that symptoms of RCCs are caused by irritation and compression effect on surrounding structures but not by invasive infiltration or endocrinological activity of the cyst tissue itself. Lu et al. concluded in their study, that an extended wall resection might lower the number of cyst recurrence [29]. This hypothesis is discussed controversially in literature, and the authors´ cohort did not reveal any confirmations of this hypothesis compared to the presented results of Chotai et al. [1, 30]. Also, it must be considered that due to the fact, that a part of the RCCs wall is part of the adenohypophysis, an entire resection is not possible without hypophyseal harm [1]. A gross total resection should therefore be sought but not be forced as it may increase the risk of postoperative endocrinological complications.

Whilst the two techniques were comparable regarding their postoperative outcome and recurrence rate, there were distinct differences in terms of complications. Especially the probability of persisting CSF fistula and consecutive rhinoliquorrhea was significantly higher in the endonasal cohort as expected due the selected surgical technique. However, the risk of intraoperative opening of the diaphragm by cyst removal and fenestration to the CSF space is much higher than in routine endoscopic intrasellar surgery [12]. This induces a higher risk of CSF fistulas in correlation to other series and pathologies treated endonasal endoscopically [12, 19, 20, 27]. The literature discuss the major disadvantage of endoscopic endonasal skull base approaches with significant risk of CSF leakage reported about 5–30% [39–44]. Although, using a sandwich-technique and lumbar drainage additionally after intraoperative CSF flow did not reduce the risk of persisting CSF fistulas in the authors´ series below the reported previous results. Therefore, some authors suggest a surgical technique of cyst fenestration in cases of RCCs via an endonasal approach [24]. In the authors´ opinion this surgical concept is not an adequate treatment because of the high recurrence rate and insufficient cyst opening. The radicality and recurrence rate of the authors´ series underlines the hypothesis that a partial resection of the cyst wall and communication of the RCC to the CSF space is necessary to prevent a recurrence in long-term follow up. An intraoperative perforation of the diaphragm with CSF flow was seen in about one half of the cases in the presented cohort. On the one hand, it paves the way for postoperative CSF fistulas and infections, but on the other hand, authors suppose that it might decrease the risk for RCC recurrence.Seen that after transsphenoidal surgery, the sphenoid bone and thereby the hypophyseal fossa will usually be closed, newly secreted cyst content might accumulate and put pressure on surrounding structures again [12, 19, 45]. A constant connection to the intracranial CSF system via a controlled opening of the diaphragm could prevent this mechanism. Further complications in the transsphenoidal cohort were nasal and endocrinological dysfunctions. These results are in line with previous reports [12–15, 27]. Previous research showed that nasal complaints could decrease patients’ quality of life significantly by limiting sleep quality and performance [14, 46, 47]. In the presented study, postinterventional treatment by an ENT physician was necessary in a fourth of the endonasal cohort. Comparable percentages were described in the literature [11, 14, 46, 47]. Additionally, the endonasal route can induce hypo- or anosmia if an extended approach is necessary for a large exploration. Postoperative endocrinological dysfunction was the most common complication in the presented cohorts. However, it has to be distinguished in to transient and permanent dysfunction. Since transient endocrinological complications were well treatable and disappeared after a few weeks, their relevance is negligible in decision-making. However, permanent dysfunction goes along with lifelong medicamentous substitution and laboratory tests and should therefore be avoided. Although endocrinological complications were more common after transsphenoidal interventions in this study, data in further literature are not consistent. Fan et al. for example presented a comparable study design with comparison of the transsphenoidal and transcranial technique in the treatment of RCCs and reported, that hormonal dysfunction appeared more often after transcranial surgery [11]. Further studies regarding both techniques in the context of other pathologies like craniopharyngeomas or meningeomas showed ambiguous results [22, 27, 48]. Apart from one case with endocrinological complications, the main risk of the transcranial approach in the authors´ study were lesions of cranial nerves: two of these 13 patients showed transient hyposmia, hypaesthesia or a limited facial expression caused by an irritation of the olfactory nerve, trigeminal nerve or the temporal branch of the facial nerve during intervention. As all patients recovered within one year, these complications did not affect their quality of life in the long-term. Peng et al., who analysed a cohort of exclusively transcranial operated patients, had only two cases of transient postoperative Diabetes insipidus, but no other complications [5]. Their study confirms the authors´ results of a low complication rate of the transcranial keyhole approach. This outcome could be attributed to the larger surgical corridor and improved maneuverability within the suprasellar space provided by the transcranial approach. Additionally, there is a lower risk of damage to the pituitary stalk and gland tissue via this route. Conversely, there is a higher risk of manipulation of the optic chiasm and optic nerves, although the authors did not observe any complications or deterioration in visual function in their series. To minimize these surgical risks and enhance surgical outcomes, the authors advocate for the use of an endoscopic-assisted microsurgical technique via a keyhole approach. They argue that many drawbacks associated with classical transcranial routes to the sellar region can be minimized using a supraorbital keyhole approach. Theoretical benefits include reduced brain retraction, smaller skin incisions with less tissue dissection, resulting in fewer postoperative complications, and a more direct approach to specific pathologies of the skull base. Another significant advantage of the supraorbital approach via an eyebrow skin incision is the wide-opened surgical field in depth, increasing the distance from the craniotomy through a small opening. Paradoxically, this can make the superior surgical field at the anterior skull base and sellar region appear larger than expected via a minicraniotomy. Furthermore, such minimally invasive approaches can address complications such as temporalis muscle atrophy, mandibular pain, and chewing difficulties postoperatively [32, 49, 50]. The major advantage of a transcranial route to RCCs is the large opening with NTR or GTR without the high risk of CSF fistulas and new hormonal deficiencies of the different axes. In addition, nasal complaints can be avoided completely via the supraorbital approach.

The endoscope serves as a crucial instrument for the successful surgical procedure using a keyhole approach. The utilization of the endoscope, along with the identification of anatomical structures and remnant tissue in the presented cohort, underscores this assertion. Endoscope-assisted techniques in the realm of neurosurgical skull base surgery have demonstrated several advantages [32, 51, 52]: they enhance illumination within the depths of the surgical field, offer a detailed depiction of anatomical features in close proximity, and provide an expanded view, also around corners, through angled telescopes. However, it’s important to note that the exposure provided by endoscopes is not three-dimensional, and surgeons must be adept with the specialized endoscopic devices and anatomical features. They also require sufficient training for eye-hand coordination in the endoscopic view. The endoscope may limit the operating range of the instruments, depending on angle and depth of the selected approach to the RCC. An area where the endoscope is probably essential for resection control is the region under the ipsilateral optic nerve for inspection of diaphragm sellae or retrochiasmatic space as well as in the optocarotid window and laterally to the internal carotid artery.

In this context, the authors want to indicate the meaning of modern technical innovations who aim to continuously ameliorate and facilitate surgical work conditions. More precise instruments will help to lower also the transsphenoidal complication rate and make these operations even safe [5, 16, 17, 28, 53].

When considering the ideal approach to a RCC in a selected case, the neurosurgeon must filter out those complications of those which were discussed, that lead to a momentous or long-lasting limitation of the patients’ quality of life. In authors´ opinion, these include CSF fistulas with necessary reoperation, permanent endocrinological dysfunction and persisting nasal complaints. All of them were more frequent or even limited to the transsphenoidal approach. In combination with the equal postoperative outcome concerning radicality, recurrence rate and visual function, the transcranial keyhole approach should be recommended if both approaches are feasible.

It must be stated, that the patient cohort is relatively small due to the low incidence of symptomatic RCCs, and that a longer follow-up period up to 10 years ore even more would give us more specific information about recurrence rates and surgical outcome of both approaches. The mean follow up time period was more than 5 years in the presented study. However, there are reported recurrences of RCCs 5–10 years after surgery in the literature in some cases. Additionally, no blinded direct comparison between both surgical approaches has been performed. The authors pointed out these limitation keeping in mind that the realisation of a blinded randomised analysis of this surgical procedure and different approaches in this pathology might be impossible. The results reflect an unavoidable personal preference of the operating neurosurgeon regarding the choice of approach in correlation to tumor size and localisation. However, the presented findings depend on the individual anatomy of the tumor. Therefore, a reevaluation of the presented data after further follow-up and further studies with more patients are necessary to substantiate the results.

## Conclusion

Although both techniques allow a safe and particularly efficient cyst resection, the authors propose the minimally invasive transcranial keyhole approach for all RCCs that are anatomically resectable via both techniques. This recommendation is conditioned on the equal postoperative outcome but probably slightlylower complication rate after transcranial operations as presented in thisstudy by the authors which were able to perform both techniques equally in their surgical setting and experienceA radical resection should not be forced, as it did not improve the outcome. Of course, also the surgeons experience and the available equipment is to be considered during decision-making as well as individual patients characteristicsFurthermore, it should kept in mind that surgical instruments are continuously being ameliorated by modern development in order to improve work conditions and lower complication rates. Additional studies with longer follow-up periods and higher numbers of cases are necessary to state the presented data more precisely and to get additional findings concerning the approaches effect on recurrence.

## Electronic supplementary material

Below is the link to the electronic supplementary material.

Supplementary Material 1: Video 1 shows details of surgical procedure of an exemplary case of Rathke´s cleft cyst resected via supraorbital keyhole approach.

Supplementary Material 2: Video 2 shows details of surgical procedure of an exemplary case of Rathke´s cleft cyst resected via endonasal approach.

## Data Availability

No datasets were generated or analysed during the current study.

## Abbreviations

ACTH: Adrenocorticotropic hormone
CSF: Cerebrospinal fluid
cCT: Cranial computer tomography
EVD: External ventricular drainage
ENT: Ear-Nose-Throat
FSH: Follicle stimulating hormone
GH: Growth hormone
GTR: Gross total resection
IGF: I-Insulin-like growth factor 1
LH: Luteinizing hormone
MRI: Magnetic resonance imaging
NTR: Near total resection
RCC: Rathke´s cleft cyst
SD: Standard definition
STR: Subtotal resection
TSH: Thyroid-stimulating hormone
